# Environmental Dissemination of Selected Antibiotics from Hospital Wastewater to the Aquatic Environment †

**DOI:** 10.3390/antibiotics9070431

**Published:** 2020-07-21

**Authors:** Mutshiene Deogratias Ekwanzala, Raisibe Florence Lehutso, Teddy Kabeya Kasonga, John Barr Dewar, Maggy Ndombo Benteke Momba

**Affiliations:** 1 Department of Environmental, Water and Earth Sciences, Tshwane University of Technology, Arcadia Campus, Private BagX680, Pretoria 0001, South Africa; ekwanzala.md@gmail.com (M.D.E.); teddykasonga@yahoo.fr (T.K.K.); 2 Water Centre, Council for Scientific and Industrial Research, Pretoria 0001, South Africa; FLehutso@csir.co.za; 3 Department of Life and Consumer Sciences, University of South Africa, Florida Campus, Johannesburg 1709, South Africa; dewarj@unisa.ac.za

**Keywords:** antibiotics, environment dissemination, UPLC-MS, wastewater, sludge, sediment

## Abstract

The environmental dissemination of selected antibiotics from hospital wastewater into municipal wastewater and lastly to a receiving water body was investigated. Selected antibiotics (azithromycin (AZM), ciprofloxacin (CIP), clindamycin (CDM), doxycycline (DXC) and sulfamethoxazole (SMZ)) present in effluents of academic hospital wastewater, influents, sewage sludge, and effluents of municipal wastewater, receiving water, and its benthic sediment samples were quantified using the Acquity^®^ Waters Ultra-Performance Liquid Chromatography System hyphenated with a Waters Synapt G2 coupled to a quadrupole time-of-flight mass spectrometer. The overall results showed that all assessed antibiotics were found in all matrices. For solid matrices, river sediment samples had elevated concentrations with mean concentrations of 34,834, 35,623, 50,913, 55,263, and 41,781 ng/g for AZM, CIP, CDM, DXC, and SMZ, respectively, whereas for liquid samples, hospital wastewater and influent of wastewater had the highest concentrations. The lowest concentrations were observed in river water, with mean concentrations of 11, 97, 15, and 123 ng/L, except for CDM, which was 18 ng/L in the effluent of wastewater. The results showed that the highest percentages of antibiotics removed was SMZ with 90%, followed by DXC, AZM and CIP with a removal efficiency of 85%, 83%, and 83%, respectively. The antibiotic that showed the lowest removal percentage was CDM with 66%. However, the calculated environmental dissemination analysis through the use of mass load calculations revealed daily release of 15,486, 14,934, 1526, 922, and 680 mg/d for SMZ, CIP, AZM, DXC, and CDM, respectively, indicating a substantial release of selected antibiotics from wastewater to the river system, where they are possibly adsorbed in the river sediment. Further research into the efficient removal of antibiotics from wastewater and the identification of antibiotic sources in river sediment is needed.

## 1. Introduction

Hospital wastewater (HW) in many low to middle income countries (LMICs) is released without any prior treatment to the municipal sewer network, despite the knowledge of the existence of highly toxic organic compounds such as endocrine-disrupting chemicals and antibiotics [1]. Antibiotics are of particular interest, as it has been shown to induce antimicrobial resistance at the sublethal level if found in the environment [2]. Antimicrobial resistance is a significant public health issue of the 21st century, and it has been predicted that it will claim up to 10 million lives yearly if adequate solutions are not implemented [3,4]. Hence, extensive research has been conducted to quantify antibiotics in the environment, particularly in municipal or urban wastewater [2,5,6,7,8,9].

Antibiotics have been quantified from different environmental matrices worldwide [10,11,12]. Numerous classes of antibiotics have been reported in HW [6,13], wastewater treatment plants (WWTP) influent (IW) and effluent (EW) [2], sludge (AS) [14], surface water (RW) [2], and water body sediments (RS) [15]. The concentrations reported are in higher µg/g to low mg/g for solid samples such as AS and sediment and in low ng/L to few µg/L for aqueous samples such as wastewater effluents and RW. Generally, HW has been reported to carry up to 150 times the concentration of antibiotics that municipal wastewater [16]. Studies reporting residual concentrations of different antibiotics in HW, IW, AS, EW, RW and RS are presented in Table 1. As it can be seen in Table 1, the current research has mainly focused on high-income countries, while for LMICs, there are little to no data reported, especially on the assessment of numerous classes of antibiotics [6]. Even in the available literature, studies relating to antibiotics have not yet investigated the concentration of antibiotics in soil fertilised with sewage sludge for growing crops.

While the quantities of antibiotics in different environmental compartments are known, especially in aquatic environments; their dissemination from HW to natural resources is poorly understood in LMICs. Currently, studies relating to antibiotics have not yet investigated the concentration of antibiotics that are removed from sewage sludge that is applied to crop fields as fertiliser, which can be found in nearby water bodies through run-off. As stated above, low concentrations have been reported from surface water bodies [25]. Furthermore, to the best of our knowledge, the environmental dissemination of multi-class antibiotics has not been investigated, and their quantity in AS and RS is not well elucidated. Although the concentrations reported are in trace levels, these have been reported to contribute to the development of antibiotic resistance; hence urgent attention needs to be directed to understanding the full spectrum of the environmental dissemination of antibiotic.

While reports from high-income countries reports low quantities of antibiotics in no HW, their presence has been shown to contribute to the development of antibiotic resistance. Hence urgent attention needs to be directed towards understanding the full spectrum of the environmental dissemination of antibiotics. Hence, the objective of the current study was to quantify the antibiotics and assess the dissemination of commonly used antibiotics from an academic hospital in Gauteng, South Africa (SA) to the receiving aquatic environment. Antibiotic selection was based on a prescription pattern consulted at a major medical aid scheme and consultation with the pharmacy department of the sampled academic hospital. The complete list with corresponding physicochemical properties of the antibiotics investigated is present in Table 2. Multi-class antibiotics, namely azithromycin (AZM), ciprofloxacin (CIP), clindamycin (CDM), doxycycline (DXC), and sulfamethoxazole (SMZ), were quantified in different matrices that channel the dissemination of antibiotics from HW through WWTP to mainly the RW matrix, to establish the dissemination trends and contribution to antibiotic pollution from different wastewater matrices. The matrices investigated included HW, IW, and EW of WWTP, municipal AS, and RS.

## 2. Materials and Methods

### 2.1. Reagents and Materials

LC-MS grade acetonitrile (ACN), methanol (MeOH), ammonium acetate, formic acid and hydrochloric acid and analytical grade disodium ethylenediaminetetraacetate (Na_2_EDTA) ACS reagent were all purchased from Sigma Aldrich (Sigma Aldrich, Johannesburg, South Africa). European Pharmacopeia standards of all selected antibiotics were purchased from LGS standards (Industrial Analytics, Johannesburg, South Africa) and are listed in Table 2 with their corresponding information. One mg/L stock solution of the standard antibiotic mixture was prepared in MeOH/dH_2_O in a 20:80 ratio (*v/v*), except for ciprofloxacin, which was prepared in MeOH containing 0.5% hydrochloric acid. A solution of ACN/2 mM ammonium acetate solution/formic acid in 3/97/0.05 (*v/v/v*) was used as mobile phase A, while mobile phase B was made up of ACN/2 mM ammonium acetate/formic acid 95/5/0.05 (*v/v/v*). Mobile phase C (ultra-pure water containing 10 mmol/L ammonium acetate/MeOH) was used to increase ionisation. Mobile phases were filtered (0.22 µm, Sigma Aldrich, South Africa) before use. All prepared standards were stored in the dark at 4 °C. Deionised water used throughout the study was obtained from a Purite RO Water Purification System (Suez Water, Lasec SA, Cape Town, South Africa). Liquid samples (HW, IW, EW, and RW) were filtered using 0.45 µm nitrocellulose filters paper (Merck, Johannesburg, South Africa) to remove debris before extraction, using Supel-Select HLB cartridges (500 mg/12 mL) from Sigma Aldrich, South Africa.

### 2.2. Sample Sites, Collection and Preparation

**Figure 1:**
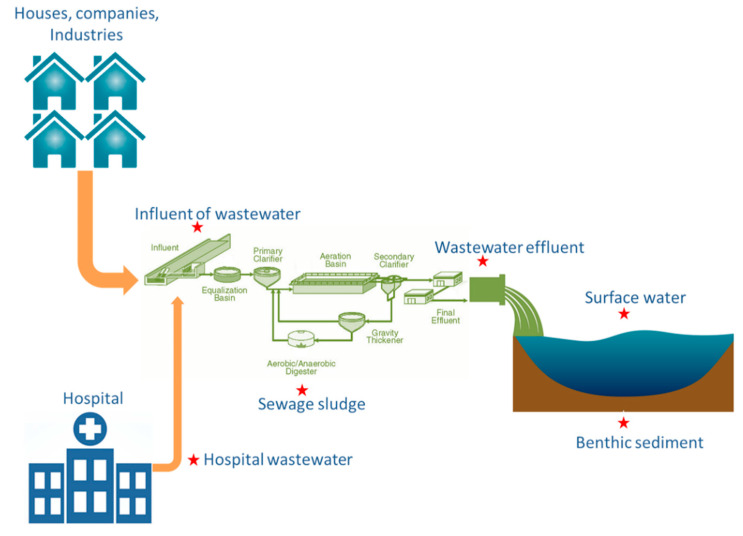
Overview of the sampling points. Stars indicate sample matrices.

HW was collected from an academic hospital in Gauteng Province, SA. IW, AS and EW were collected from the HW-receiving municipal WWTP. In addition, receiving RW and its RS, as illustrated in Figure 1 were also collected. The selected academic hospital, situated in the City of Tshwane, Gauteng, SA, has more than 1077 beds for the hospitalisation of inpatients afflicted with various diseases. Its receiving municipal WWTP, also located in the City of Tshwane, Gauteng, SA, has built a capacity of 51 megalitres per day and serves more than 40,000 residents. This wastewater plant employs technologies such as biological nutrient removal through activated sludge and biological filters for liquid processing, whereas sludge treatment is achieved through dissolved air flotation thickening, anaerobic digestion, and solar drying beds.

Amber bottles were washed with formic acid, MeOH and dH_2_O and wrapped in aluminium foil before use. Samples collected as part of a 24-h composite sample were collected on arrival at the municipal wastewater plant from the IW, AS, and EW compartments. For the sampling of HW and RW, a method previously used by Ekwanzala et al. [26,27] was followed, where one sample consisted of a composite of five grab samples collected at 30-min intervals for better environmental representativeness of the matrix sampled. RW samples were collected within 100 m of the WWTP outlet (EW). For RS samples, surface bed sediment from the top 2 cm was collected with a flat hand shovel as sub-samples from several points with low current velocities, in order to obtain the finest-grained sediment. Thus, RS settled during low and medium discharge were sampled. Four sampling campaigns were conducted on 15 May, 10 June, 18 September, and 6 November 2018. Prior to sampling, 10% formaldehyde was added to the sampling bottle to preserve the antibiotics from degradation, and 500 mL of water samples was spiked with 0.8 mg/mL of Na_2_EDTA to prevent metal ions that could hinder DXC recovery. During every sampling, three true replicates (~1 L each) were sampled in amber bottle containers. The samples were immediately taken to the laboratory on ice packs (4 °C) for analysis. Upon arrival, samples were then manually shaken and filtered through 0.45 µm nitrocellulose membrane filter paper to remove debris and then stored in the dark at 4 °C.

Fifty grams of AS and RS were sequentially extracted with 45 mL of acetone/acetic acid in a 20/1 (*v/v*) and 3 × 45 mL ethyl acetate. In each event, the slurries were hand-shaken, sonicated for 25 min at 30 °C and centrifuged (4000 rpm). The supernatant from the four extraction events was then combined and concentrated to 1–3 mL using a Buchi rotary evaporator (Labotec, Johannesburg, South Africa). The sample’s pH was adjusted to 3.5 for optimal extraction of antibiotics in the solid phase extraction (SPE), as described by Rossmann et al. [17].

### 2.3. Solid-Phase Extraction

HW, IW, EW, and RW were extracted using SPE (Supel-Select HLB cartridges, a hydrophilic-lipophilic-balanced reversed-phase sorbent set on a vacuum 12-position SPE Extraction Manifold from SupelCo—Sigma Aldrich, South Africa) for the extraction of the selected antibiotics from wastewater. The extraction method using the Supel-Select HLB cartridges was adapted as optimised by Rossmann et al. [17], with slight modification. Briefly, Supel-Select HLB cartridges were conditioned with 3 × 3 mL of MeOH/dH_2_O/formic acid in a 90/9/1 (*v/v/v*) followed by 3 × 3 mL of dH_2_O and 3 × 3 mL of 10 mmol/L of Na_2_EDTA. Samples were then passed through the conditioned SPE-cartridge at a flow rate of 3 mL/min. Thereafter, the cartridge was washed using 3 mL of dH_2_O and vacuum air-dried for 5 min. Finally, compounds were eluted using 3 × 2 mL of MeOH/dH_2_O/formic acid 90/9/1 (*v/v/v*). Eluates were evaporated to dryness using nitrogen at room temperature and reconstituted with 250 µL of mobile phase A and B (125 µL each). Analysis was carried out using UPLC-MS, as described below.

### 2.4. Sample Analysis

Selected antibiotics were quantified using the Acquity^®^ Waters Ultra-Performance Liquid Chromatography System hyphenated with a Waters Synapt G2 coupled to a quadrupole time-of-flight mass spectrometer (UPLC-QTOF-MS). A reversed-phase Waters UPLC^®^ C18 Ethylene Bridged Hybrid 1.7 µm particle size (2.1 mm ID × 100 mm length) column (incubated at 30 °C) was used for chromatographic separation, where 10 µL of the extracts was injected. The antibiotic separation was performed using a binary mobile phase, mobile phases A and B. A gradient elution programme at a flow rate of 0.2 mL/min without any split was developed. The gradient elution started with mobile phase A, was maintained for 1 min, and then increased with a linear gradient from 20% to 50% within 1 min and 80% over the next 3 min with a curve-2 gradient. Subsequently, the amount of mobile phase B was lowered to 20% in 3 min and maintained for the next 5 min. The system was equilibrated (mobile phases A and B, 50/50) before the next run.

Direct flow and scan mode were used to determine the precursor ions from the 0.01 ng/L standards mixture. Each antibiotic was then optimised to generate product ions. Instrumental parameters were optimised and are indicated in Table 3 and Table 4. Data acquisition was carried out in selected reaction monitoring mode, and data processing was done with MassLynx™ (v4.1) Mass Spectrometry Software (Waters, Milford, MA, USA). Detection was done in a HESI+ mode.

### 2.5. Statistical Analysis

**Figure 2:**
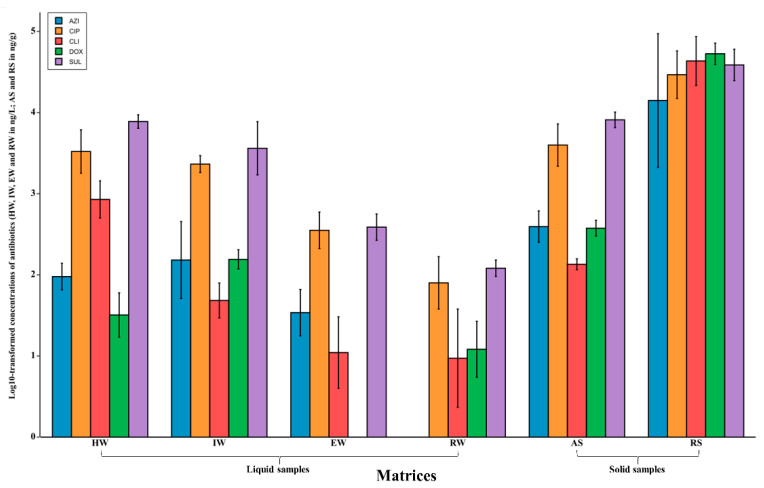
Concentrations of targeted antibiotics in the assessed matrices as arithmetic means (columns) with error bars indicating the range of results; HW, IW, EW and RW were quantified in ng/L, whereas AS and RS were quantified in ng/g.

Antibiotic concentrations detected from different environments and dates were exported to Microsoft Excel and then transferred into BioVinci Software (BioTuring, San Diego, CA, USA) for statistical analysis. For better visualisation across matrices, antibiotic concentrations detected in ng/L were log_10_ transformed and visualised in Figure 2. Two-way ANOVA (using sampled dates and antibiotic concentrations as variables) and Spearman’s correlation (correlations were monotonic, not linear) were applied to antibiotic concentrations to compare and investigate the correlation between assessed matrices. Furthermore, a two-way ANOVA with a Tukey HSD post hoc test (honest significant difference) was performed to assess the difference in collection dates. A *p* value was Bonferroni corrected and considered statistically significant if less than or equal to 0.05.

## 3. Results

### 3.1. Calibration Curve and Quality Assurance

All parameters related to the method development, calibration curve, validation, sensitivity, accuracy and precision are presented in Table 3 and Table 4. The calibration was conducted from 10 to 2000 ng/L (8 points) using the working standard solutions in MeOH-dH_2_O (20:80, *v/v*) containing all the target antibiotics. The correlation coefficients (R^2^) of the obtained calibration curves of all the target compounds ranged from 0.9902 to 0.9959 (Table 3). The limit of blank (LoB) assessing matrix effects was calculated as per Equation (1). The limit of detection (LoD), defined as the concentration that corresponds to three times the standard deviation of blanks, was measured using Equation (2) below. The limit of quantification (LoQ) was determined as ten times the LoD, and it was calculated using Equation (3). Low LoQs were achieved, ranging from 0.3 to 3.70 ng/L for the target antibiotics (Table 4). The recovery of the target compounds was carried out by spiking three replicates of river water samples with standard antibioticss at 50, 200, and 500 ng/L, respectively, with blank subtraction. The recovery of the antibiotics generally exceeded 75%, and the relative standard deviations were less than 20% (Table 4). Both parameters were calculated using Equations (4) and (5), respectively. Spiked matrices (no less than 50% of the number of samples) were treated and analysed to determine the recoveries during the measurements to guarantee the precision of quantification. Quantification was based on peak areas of selected ion chromatograms on target parent ions, product and retention time. Procedure blanks, solvent blanks, and quality standards were also analysed, alongside the measurements to monitor the performance of the method.

LoB = mean_blank_ + 1.645(SD_blank_) (1)

(2) $$\mathrm{LoD}=\frac{\mathrm{Lowest}\;\mathrm{concentration}\;\mathrm{of}\;\mathrm{standard}}{\mathrm{Instrumental}\frac{S}{N}\mathrm{for}\;\mathrm{lowest}\;\mathrm{concentration}\;\mathrm{of}\;\mathrm{standard}}\times 3$$

(3) $$\mathrm{LoQ}=\frac{\mathrm{Lowest}\;\mathrm{concentration}\;\mathrm{of}\;\mathrm{standard}}{\mathrm{Instrumental}\frac{S}{N}\mathrm{for}\;\mathrm{lowest}\;\mathrm{concentration}\;\mathrm{of}\;\mathrm{standard}}\times 10$$

(4) $$\%\;\mathrm{Recovery}=\frac{\mathrm{Intrument}\;\mathrm{quantification}}{\mathrm{Standard}\;\mathrm{spiked}\;\mathrm{concentration}}\times 100$$

(5) $$\%\;\mathrm{RSD}=\frac{\mathrm{Standard}\;\mathrm{deviation}}{\mathrm{mean}}\times 100$$

### 3.2. Antibiotics Quantities

Twenty-four composite pooled samples were collected during the sampling procedures, and the log_10_ transformed concentrations of all assessed antibiotics across different matrices are displayed on bar plots (Figure 2). Overall, all assessed antibiotics were found in all matrices with detection frequencies of 98%, 100%, 100%, 93%, and 100% for AZM, CIP, CDM, DXC, and SMZ, respectively. For solid samples, RS had the highest concentrations as compared to AS, with mean concentrations of 34,834 (4.54 log_10_), 35,623 (4.55 log_10_), 50,913 (4.71 log_10_), 55,263 (4.74 log_10_), and 41,781 ng/g (4.62 log_10_) for AZM, CIP, CDM, DXC, and SMZ, respectively. For liquid samples, the lowest concentrations were observed in RW, with mean concentrations of 11 (1.07 log_10_), 97 (1.99 log_10_), 15 (1.19 log_10_), and 123 ng/L (2.09 log_10_), except for CDM, which was 18 ng/L (1.26 log_10_) in EW. IW had mean concentrations in the same order of magnitude as did HW with 247 (2.39 log_10_), 2379 (3.37 log_10_), 53 (1.73 log_10_), 160 (2.21 log_10_), and 4440 ng/L (3.65 log_10_) for AZM, CIP, CDM, DXC, and SMZ, respectively. Lastly, EW also had considerably higher mean concentrations of the assessed antibiotics compared to RW [40.58 (1.60 log_10_), 397 (2.60 log_10_), 18 (1.26 log_10_), 24 (1.39 log_10_), 411 ng/L (2.61 log_10_)]. The two-way ANOVA is illustrated in Supplementary Figure S1. This analysis shows a significant *p*-value for antibiotic concentrations (*p* = 0.000429), which indicates that the concentration of the residue of antibiotics was significantly different across matrices. The *p*-value for the interaction between antibiotics concentration and sampled dates was also significant (*p* = 0.02), indicating that the concentrations of antibiotic in assessed samples were significantly different across different sampled dates.

The Spearman’s correlation among assessed matrices that was performed is visualised in Supplementary Figure S2. Significantly positive correlations were found between the quantities of antibiotics in HW-RW (r^2^ = 0.71), HW-EW (r^2^ = 0.72), HW-AS (r^2^ = 0.59) and HW-IW (r^2^ = 0.62). The correlation between the quantities of the assessed antibiotic concentrations in IW against other matrices also showed positive correlations [IW-RW (r^2^ = 0.70), IW-EW (r^2^ = 0.74) and IW-AS (r^2^ = 0.89)]. The correlations between the quantities of antibiotics in AS against other matrices were also positive (AS-RW, AS-EW, and EW-RW). These positive correlations indicate a direct relationship between the two assessed matrices, e.g., if the antibiotic concentrations in IW were high, they were also high in the associated positively related matrix. When plotting RS quantities against other matrices, the correlation coefficients were found to be inverse and low [RS-HW (−0.19), RS-IW (−0.19), RS-AS (−0.16), RS-EW (−0.16), and RS-RW (−0.19)]. Supplementary Figure S2 indicates that the concentrations in RS are not directly proportional to other assessed matrices.

To assess the treatment plant removal efficiency, Equation (6) was used:

(6) $$\mathrm{Removal}\;\mathrm{efficiency}\;\%=\frac{\mathrm{mean}\;\mathrm{concentration}\;\mathrm{IW}\;–\mathrm{mean}\;\mathrm{concentration}\;\mathrm{EW}}{\mathrm{Mean}\;\mathrm{concentration}\;\mathrm{of}\;\mathrm{IW}}\times 100\;\;\;\;$$

Results showed that the highest removed antibiotics were SMZ with 90%, followed by both DXC, AZM and CIP with a removal efficiency of 85%, 83%, and 83%, respectively. The antibiotic that showed the lowest removal efficiency was CDM with 66%.

To assess the environmental dissemination of selected antibiotics from the WWTP to the aquatic environment, mass loads defined as the mass of pollutant being discharged from the wastewater per day were calculated as described by Kibambe et al. [28,29]. Therefore, the mass loads of selected antibiotics were determined using Equations (7) and (8) below:

Mass load of antibiotics in EW (mg/d) = C_EW_ × Q_EW_ (7)

Q_EW_ = Q_IW_ − Q_WAS_ (8)

where:

C_IW_: concentration of the antibiotic in the IW (mg/ML)

C_EW_: concentration of the antibiotic in the EW (mg/ML)

Q_EW_: flow rate of the effluent (ML/d)

Q_IW_: influent flow rate (ML/d)

Q_WAS_: waste activated sludge flow rate (ML/d)

The Q_EW_ was calculated to be 37.618 ML/d, based on an average influent flow rate of 38 ML/d, sewage sludge wasting rate of 0.382 ML/d. The results of the environmental dissemination revealed that the highest mass load of antibiotics disseminating from EW to RW was found to be SMZ with an average of 15,486 mg/d, CIP followed this with an average of 14,934 mg/d. The disseminating mass load of AZM was found to be 1526 mg/d, whereas the disseminating mass load of 922 mg/d was determined for DXC. The antibiotic which showed the lowest disseminating mass load was CDM with an average of 680 mg/d.

## 4. Discussion

The occurrence and detection of clinically relevant antibiotics in wastewater and surface have been extensively explored [9,20,30,31]. However, the trail of environmental dissemination of antibiotics used in the hospital to the aquatic environment is poorly understood, since many reports do not consider RS or AS when assessing antibiotic residue levels in environmental matrices. Concerning AS, it is a common practice worldwide to use dried AS (compost) as fertiliser on crop soil. In fact, recent studies have shown the enrichment of agricultural soil with antibiotic-resistant bacteria and genes after application of AS [32,33]. It stands to reason that antibiotics may also be transferred to such soil selecting for antibiotic-resistant bacteria and promoting horizontal gene transfer among soil microcosms. Thus, its assessment should shed light on the transfer of antibiotics from WWTP to agricultural soils. On the other hand, when it comes to RS, recent reports have highlighted a high occurrence of resistant bacteria and genes [26,27]. Knowing that riverbed sediment can resuspend and re-enter the water column [34], it is important to assess such a vital component of the RW system. In the light of the above, this study investigated the environmental dissemination of some selected frequently used antibiotics (AZM, CIP, CDM, DXC and SMZ) from HW to the aquatic environment, considering both AS and RS. Therefore, the current research article contributes to the existing literature by (i) determining the quantities of selected antibiotics in AS and RS; (ii) establishing the dissemination of antibiotics in liquid and solid matrices and (iii) establishing the antibiotic relationship among assessed matrices. Due to the lack of the exact quantity used per day or month, the correlation between antibiotic use and dissemination could not be established.

Antibiotics were detected in all assessed matrices of this study, which is consistent with other published studies [6,9,15,17,20,22,23,24], except for AS that is quantified for the first time in our study (Table 1). The antibiotic concentrations in HW in this current study were slightly higher than those reported in other countries. It has been shown that the concentrations of antibiotics in HW are directly proportional to antibiotic use in the hospital [6,22]. This can consequently explain the different variations of antibiotic concentrations found in this study compared to others. In a country devastated by two notable pandemics, human immunodeficiency virus, acquired immunodeficiency syndrome and tuberculosis, this might explain the use of antibiotics and their subsequent occurrence in the environment. Furthermore, SA has been listed as the 23rd highest consumer of antibiotics worldwide [35]. Gros et al. [9] reported a concentration of AZM and CDM in HW, as in Table 1, which is comparable with the mean concentrations of the same antibiotics found in this study. However, the concentration of CIP reported by Gros et al. [9] was higher than the mean concentration found in our study. In this study, the concentration of SMX 7884.50 ng/L is significantly lower than that reported by Brenner et al. [36]. In our study, the lowest frequency of detection was from DXC, and the detected mean concentration was low compared to that reported by Pena et al. [37]. In SA, DXC is mostly used as a prophylactic treatment for malaria. However, the sample area is not a malaria zone, which can explain its low detection frequency rate.

The average detected mean concentration of CIP in IW was found to be congruent to the quantities reported in other studies [9,17,18]. CIP was found to be the most extensively explored antibiotic among all assessed antibiotics in IW (Table 1). Two South African studies, in particular, reported two different mean concentrations found in IW. Kanama et al. [22] reported a lower concentration compared to the one reported by Agunbiade and Moodley [20]. The difference in concentration could be due to the frequency of antibiotics used, the WWTP process and population size. Furthermore, Kanama et al.’s [22] IW consisted of HW only, whereas in the current study and that of Agunbiade and Moodley [20] IW included wastewater from HW and households. In other countries, the concentration of CIP was found to be lower (Table 1) than in our study. Such a high concentration could be attributed to the high use of CIP in developing countries, as forecast by Green and Tillotson [38], who stated that CIP could be misused, leading to antibiotic pollution and resistance in microorganisms. Minimal concentrations of CDM were reported in Spain, but Germany recorded the same magnitude of concentration found in the current study. In a study conducted in Spain, DXC was not detected [9]. However, in the current study and a study conducted in Germany, it was found as highlighted in Figure 2 and Table 1, respectively. Apart from a study in SA, which reported a higher concentration of SMX in IW [24], further emphasising the country’s high SMX concentration in IW, other studies (Table 1) reported a mean concentration lower than the one detected in our study.

A limited number of studies [32,39] have reported antibiotic occurrence in wet AS. However, many studies are assessing the quantity of dried AS (compost), e.g., [40,41]. These studies have highlighted the enrichment of agricultural soil with antibiotic-resistant bacteria and genes to soil-applied AS over a prolonged period. Thus, the reported antibiotic quantity might also select for resistant microorganisms when applied to agricultural soil. As highlighted in the results section, the reported AS mean concentrations were slightly higher than concentrations found in IW. This might be due to low solubility organics, thus promoting organics to be adsorbed in AS [42]. These concentrations will subsequently be reduced in solar drying beds when AS is transformed to compost before application to agricultural soil [43]. However, sublethal concentrations are being reported to induce resistance in pathogenic and commensal microorganisms [40,41]. Since sewage treatment with activated sludge relies on biodegradation by microbial organisms, further study is needed to decipher if these reported concentrations and some high predictive concentrations may affect the functioning of AS and removal efficiency.

The EW mean concentrations found in this study were as follows: 40 (AZM), 397 (CIP), 18 (CDM), 24 (DXC), and 411 (SMZ) ng/L. Reported concentrations of AZM elsewhere were higher than those found in our study. Studies in Spain and Germany reported similar AZM concentrations in EW. Concerning CIP concentrations in EW, only studies from South Africa [20] and Vietnam [5] reported higher concentrations than our study. Studies from China, Australia, and Spain reported lower concentrations than reported in this study. It has been reported that higher-income countries tend to have lower concentrations of antibiotics in EW than LMICs due to their strict prescription policy on the use of antibiotics and proper running of WWTP [44]. When assessing the concentration of CDM, the mean concentration in this study was lower than those reported in other studies (Table 1). Although in both Spain and Germany [9,17] DXC was not found in EW, in the current study, a mean concentration of 24.5 ng/L was found. In this study, a mean concentration of 411.67 ng/L was found for SMZ. However, in another study in SA, SMZ was not detected in EW [24]. The only study to report a higher concentration for SMZ was from Vietnam. Other studies reported lower concentrations (Table 1) than observed in this study.

Different factors, such as octanol/water partition coefficient and adsorption ability, affect the removal efficiency of antibiotics through WWTP compartments [45]. In addition to this, other physicochemical parameters, including molecular mass, solubility in water, pKa, and vapour pressure, may significantly influence their removal in WWTP. As per their pKa values (Table 2), most pharmaceutical compounds, including antibiotics, are considered weak acids and/or bases. Polar nature, polycyclic aromatic hydrocarbon structures and occurrence of multifunctional groups (Table 2) can also enable antibiotics to diffuse across treatment processes [46,47]. Most antibiotics settle out in the settling tank. However, subsequent removal of antibiotics occurs through biodegradation and transformation [30]. In terms of removal rate between IW to EW, we observed a removal efficiency of 83%, 83%, 66%, 85%, and 90% for AZM, CIP, CDM, DXC, and SMZ, respectively. These figures are comparable to those reported by others that also used biological nutrient removal treatment plants [6,9,15,17,20,22,23,24]. Removal efficiency is affected by several factors. In a review by Polesel et al. [48], factors influencing the removal of antibiotics were identified as (i) retransformation of the antibiotic to its parent pharmaceuticals form, for example, conjugated metabolites and analogues, (ii) solid retention time, (iii) fractions adsorbed onto solids, and (iv) dynamics in influent and effluent loading. Such factors, as well as those mentioned above, might also have played a role in the removal of antibiotics in the current study. In our attempt to achieve 100% removal efficiency, further studies on green chemical technologies and bioprospection of suitable microbial candidates for effective removal of selected antibiotics are needed.

While the occurrence of antibiotics in RW systems has been reported extensively (Table 1), studies assessing their dissemination from HW are scarce. As highlighted in the current study, antibiotic concentrations found in RW were comparable to those found in China by Zhou et al. [19]. When relating the antibiotic concentrations in RW and EW, studies [49,50,51] have reported that sublethal concentration of therapeutic antibiotics may select for antibiotic resistance in commensals and pathogenic microorganisms in the environment. It stands to reason that the RW might be acting as a diluting medium for the incoming EW, thus explaining the decrease in antibiotic concentrations in RW.

The RS compartment exhibited elevated concentrations. Similar concentrations have been reported elsewhere [15,20,52,53]. This calls for further investigation to reveal the source of such elevated concentrations of antibiotics. In this study, such high concentrations might be attributed to gradual dissemination from EW and surrounding runoff that gets adsorbed onto RS [54,55]. The first plausible explanation of such elevated concentrations of antibiotics in RS might originate from the occasional direct release of raw sewage into freshwater by many LMICs, as reported by another study [56]. As in AS, antibiotics with low solubility get adsorbed further in RS. Thus, the elevated concentration found in RS could also be attributed to the gradual dissemination of used antibiotics in RS. The second plausible explanation is surface runoff from farming areas around the RW transporting such antibiotics used in animal and crop production [55], which then get absorbed onto RS.

In terms of environmental dissemination, as highlighted in the results section, considerable mass loads of antibiotics were found to be disseminating from WWTP to the aquatic environments and subsequently might be adsorbed onto RS. The normalised mass load reported in this study when normalised to 1000 habitants as per Verlicchi et al. [57]. The mass loads per 1000 habitants per day were lower than those reported by Verlicchi et al. [57], except for CIP (44 mg/1000 inh/d) and SMZ (79 mg/1000 inh/d), which were in the same order of magnitude as calculated by Verlicchi et al. [57]. Furthermore, the reported mass load can explain the transfer of antibiotic residues from WWTP to RW. However, the concentrations in the aquatic environment may not solely be attributed to WWTP, as possible other sources of antibiotics pollution have been reported in the literature. In fact, studies by Almakki et al. [54] and Manyi-Loh et al. [55] stated the importance of urban runoff in the hydrologic transfer of antibiotics from areas with anthropogenic sources. Thus, runoff also carries antibiotics applied in crop and animal farm production [58]. Antibiotics in runoff might come from agricultural soil and farm production, as it has been reported that 80% of antibiotics produced worldwide are directly used in animal agriculture for the prevention of infection and as growth promoters [59]. The use of antibiotics in agriculture and animal production in SA has also been reported [60], and highlights another possible source of the detected antibiotics. This might explain the high presence of antibiotic concentration, considering that the entire catchment is populated with farms. Another contributor to antibiotic pollution in freshwater might be the direct release of antibiotics by pharmaceutical companies [61]. However, in the current study, there was no pharmaceutical company in the catchment where samples were taken.

## 5. Conclusions

In conclusion, high variations were observed among assessed antibiotics across all assessed matrices. The assessed sewage treatment plant was able to remove 83%, 83%, 66%, 85%, and 90% of AZM, CIP, CDM, DXC, and SMZ, respectively. Of the assessed matrices, the RS matrix carried a considerable concentration of antibiotics, followed by HW, AS, IW, and EW. The lowest concentrations of antibiotics were detected in RW. The environmental dissemination analysis using mass load calculations revealed a substantial release of selected antibiotics from EW to the river system, where they are possibly adsorbed in the RS over a period of time. Since observed concentrations in different matrices can select for antibiotic resistance in commensals and pathogenic bacteria, as reported previously, further studies on the bioprospection of antibiotic degraders and green chemistry technologies in the WWTP are needed to curb the environmental dissemination of antibiotics.

## Figures and Tables

**Table 1:** Selected studies reporting residual concentrations of targeted antibiotics in hospital wastewater, wastewater influent, wastewater effluents, sewage sludge and benthic sediments of receiving water bodies.

Antibiotics	Concentrations in ng/L or ng/g for RS	Matrix	Country	References
Azithromycin (AZM)	85–113	HW	Spain	[9]
	ND-437	IW		
	225–592	EW		
	285	IW	Germany	[17]
	277	EW		
	690.5	IW	Tunisia	[18]
	135.45	EW		
	ND-67	RW	China	[19]
Ciprofloxacin (CIP)	13,600–139,000	RS (in ng/g)	South Africa	[20]
	14,300	RW		
	27,100	IW		
	14,100	EW		
	222	IW	China	[2]
	104.6	EW		
	90	IW	Australia	[21]
	132.2	EW		
	41.5	RW		
	5329–7494	HW	Spain	[9]
	185–613	IW		
	ND-147	EW		
	422	IW	Germany	[17]
	146	EW		
	990	HW	South Africa	[22]
	4280	HW	Vietnam	[6]
	2150	EW		
Clindamycin (CDM)	184–1465	HW	Spain	[9]
	14–37	IW		
	18–57	EW		
	41	IW	Germany	[17]
	151	EW		
Doxycycline (DXC)	ND	HW	Spain	[9]
	ND	IW		
	ND	EW		
	10–20	RW	USA	[15]
	13,000–27,600	RS (in ng/g)		
	259	IW	Germany	[17]
	ND	EW		
Sulfamethoxazole (SMZ)	60–80	RW	USA	[15]
	12,000–17,000	RS (in ng/g)		
	98–2200	IW	USA	[23]
	140	RW		
	304	IW	China	[2]
	100.4	EW		
	65–200	HW	Spain	[9]
	ND-528	IW		
	19–198	EW		
	35,000	IW	South Africa	[24]
	ND	EW		
	515	IW	Germany	[17]
	191	EW		
	365.5	IW	Tunisia	[18]
	126.7	EW		
	9800	HW	Vietnam	[6]
	6400	EW		

ND: Not detected; HW: hospital wastewater; IW: influent of wastewater; EW: effluent of wastewater; RW: river water; RS: river sediment. Studies reporting residual concentrations of different antibiotics in hospital wastewater, wastewater influent, wastewater effluents, sewage sludge and benthic sediments of receiving water bodies.

**Table 2:** General physicochemical properties of selected antibiotics.

Antibiotics	Class of Antibiotics	Molar Mass (g/mol)	pKa Values ^a^	Structures
AZM	Macrolide	748.984	12.43, 9.57	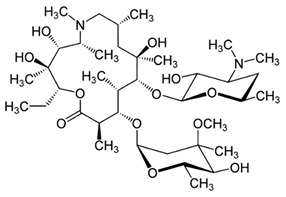
CIP	Quinolone	331.346	5.76, 8.68	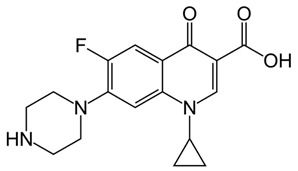
CDM	Lincosamide	424.98	5.91, 6.74	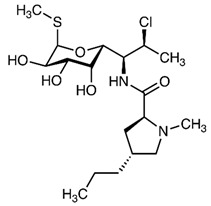
DXC	Tetracycline	444.43	−2.2, 7.75	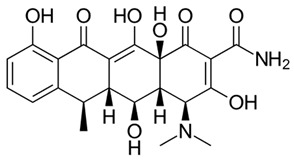
SMZ	Sulphonamide	253.279	6.16, 1.97	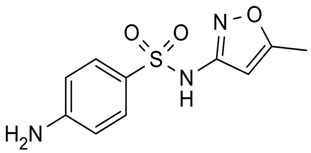

pKa: acidic or basic dissociation constant; AZM: azithromycin; CIP: ciprofloxacin; CDM: clindamycin; DXC: doxycycline; SMZ: sulfamethoxazole; ^a^ Source: www.chemaxon.com/products/calculator-plugins/property-predictors; Major physicochemical properties of the selected antibiotics.

**Table 3:** Mass spectrometric parameters for detection.

Antibiotics	m/z	Products Ions	Rt/min	Linear-Range ng/L
AZM	748.98	749.51	7.16	10–2000
CIP	331.34	332.14	3.23	10–2000
CDM	424.98	425.18	4.49	10–2000
DXC	444.43	443.14	4.47	10–2000
SMZ	253.27	254.05	1.43	10–2000

m/z: mass per charge number of ions, Rt/min: retention tine per minute.

**Table 4:** Method validation parameters.

Antibiotics	r^2^	Regression Equation	LoD (ng/L)	LoQ (ng/L)	Mean Recovery (%)
AZM	0.9959	y = 2.2261x − 15.562	0.2 × 10^−5^	0.8 × 10^−4^	94.1 ± 4.1
CIP	0.9902	y = 0.9879x − 46.064	13.5 × 10^−5^	45.0 × 10^−4^	97.4 ± 7.4
CDM	0.9948	y = 3.6348x + 230.9	1.1 × 10^−5^	1.4 × 10^−4^	95.4 ± 5.4
DXC	0.9914	y = 1.6894x + 493.81	8.8 × 10^−5^	29.2 × 10^−4^	95.3 ± 15.3
SMZ	0.9902	y = 1.6286x + 119.91	5.1 × 10^−5^	17.1 × 10^−4^	95.2 ± 5.2

r^2^: correlation coefficient, LoD: limit of detection, LoQ: limit of quantification.

## Supplementary Materials

The following are available online at https://www.mdpi.com/2079-6382/9/7/431/s1, Figure S1: Two-way ANOVA visualisation of data interactions between assessed antibiotics (AZM, CIP, CDM, DXC and SMZ) and sampled dates on HW (A), IW (B), AS (C), EW (D), RW (E) and RS (F), Figure S2: Spearman correlation heat map with correlation coefficient among assessed matrixes.
