# Supplementary material for: Environmental Dissemination of Selected Antibiotics from Hospital Wastewater to the Aquatic Environment
Source: Antibiotics (Basel). 2020 Jul 21;9(7):431. doi: 10.3390/antibiotics9070431 (PMC7400012; doi:10.3390/antibiotics9070431)
Supplement: Supplementary file 1 [file antibiotics-09-00431-s001.pdf]

antibiotics

Supplementary information for Ekwanzala et al. 2020_Antibiotics Quantification

Running title: Environmental dissemination of antibiotics

Mutshiene Deogratias Ekwanzala [1], Raisibe Florence Lehutso [2], Teddy Kabeya Kasongo [1], John Barr Dewar [3] and Maggy Ndombo Benteke Momba [1,*]

[1] Department of Environmental, Water and Earth Sciences, Tshwane University of Technology, Arcadia Campus, Private BagX680, Pretoria 0001, South Africa; ekwanzala.md@gmail.com (M.D.E.); teddykasonga@yahoo.fr (T.K.K.)

[2] Water Centre, Council for Scientific and Industrial Research, Pretoria, South Africa

[3] Department of Life and Consumer Sciences, University of South Africa, Florida Campus, Johannesburg 1709, South Africa; dewarj@unisa.ac.za

* Correspondence: mombamnb@tut.ac.za; Tel.: +27123826365

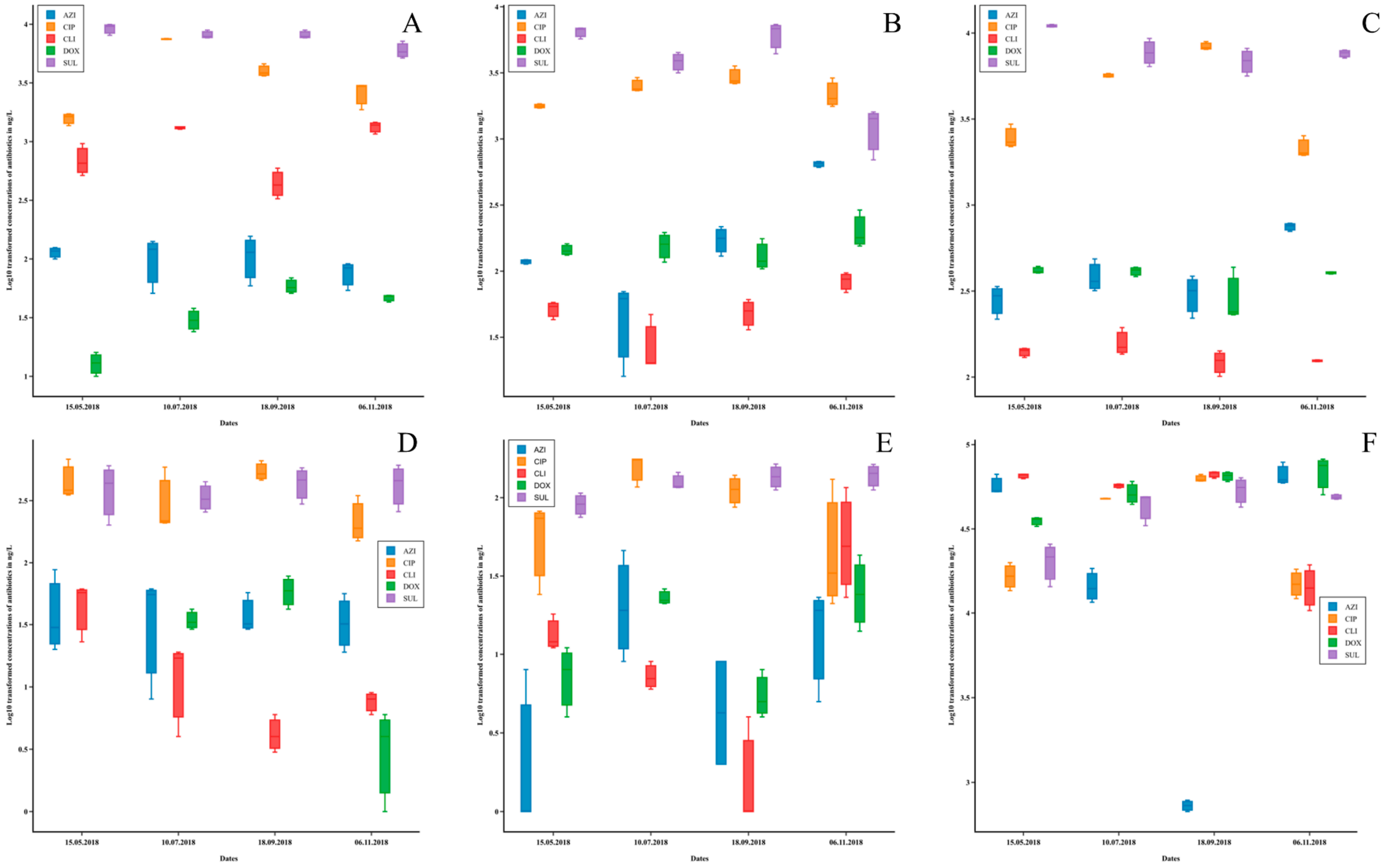

Figure 1. Two-way ANOVA visualisation of data interactions between assessed antibiotics (AZM, CIP, CDM, DXC and SMZ) and sampled dates on HW (A), IW (B), AS (C), EW (D), RW (E) and RS (F).

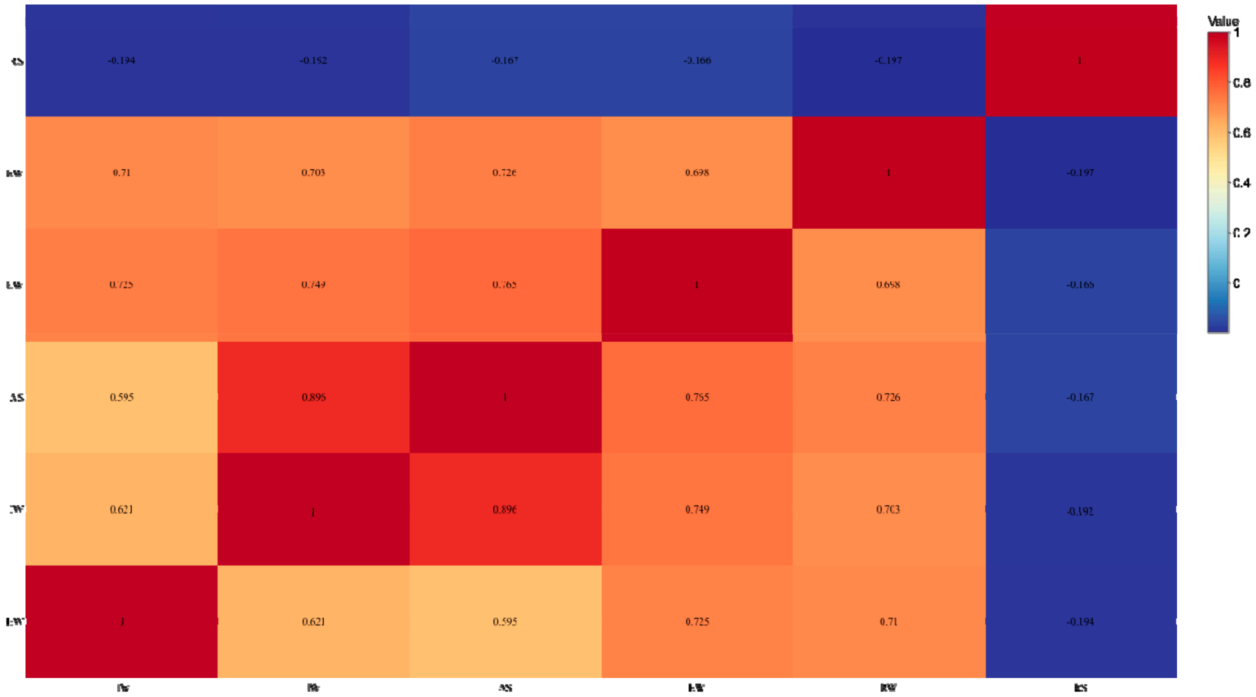

Figure 2. Spearman correlation heat map with correlation coefficient among assessed matrixes.
